# Prevalence and Economic Burden of Respiratory Diseases in Central Asia and Russia: A Systematic Review

**DOI:** 10.3390/ijerph17207483

**Published:** 2020-10-14

**Authors:** Aizhamal Tabyshova, Berik Emilov, Maarten J. Postma, Niels H. Chavannes, Talant Sooronbaev, Job F. M. van Boven

**Affiliations:** 1 Pulmonology Department, National Center of Cardiology and Internal Medicine named after M.M. Mirrakhimov, Bishkek 720040, Kyrgyzstan; emilov9090@mail.ru (B.E.); sooronbaev@yahoo.com (T.S.); 2 Unit of Global Health, Department of Health Sciences, University Medical Center Groningen, University of Groningen, 9700 RB Groningen, The Netherlands; m.j.postma@rug.nl; 3 Department of Economics, Econometrics & Finance, Faculty of Economics & Business, University of Groningen, 9700 AV Groningen, The Netherlands; 4 Department of Pharmacology & Therapy, Airlangga University, Surabaya 60115, Indonesia; 5 Department of Public Health and Primary Care, Leiden University Medical Center, 2333 ZA Leiden, The Netherlands; n.h.chavannes@lumc.nl; 6 Groningen Research Institute for Asthma and COPD (GRIAC), University Medical Center Groningen, University of Groningen, 9700 RB Groningen, The Netherlands; j.f.m.van.boven@umcg.nl

**Keywords:** prevalence, economic burden, chronic respiratory diseases, Central Asia, Russia

## Abstract

Prevalence data of respiratory diseases (RDs) in Central Asia (CA) and Russia are contrasting. To inform future research needs and assist government and clinical policy on RDs, an up-to-date overview is required. We aimed to review the prevalence and economic burden of RDs in CA and Russia. PubMed and EMBASE databases were searched for studies that reported prevalence and/or economic burden of RDs (asthma, chronic obstructive pulmonary disease (COPD), cystic fibrosis, interstitial lung diseases (ILD), lung cancer, pulmonary hypertension, and tuberculosis (TB)) in CA (Kyrgyzstan, Uzbekistan, Tajikistan, Kazakhstan, and Turkmenistan) and Russia. A total of 25 articles (RD prevalence: 18; economics: 7) were included. The majority (*n* = 12), mostly from Russia, reported on TB. TB prevalence declined over the last 20 years, to less than 100 per 100,000 across Russia and CA, yet in those, multidrug-resistant tuberculosis (MDR-TB) was alarming high (newly treated: 19–26%, previously treated: 60–70%). COPD, asthma (2–15%) and ILD (0.006%) prevalence was only reported for Russia and Kazakhstan. No studies on cystic fibrosis, lung cancer and pulmonary hypertension were found. TB costs varied between US$400 (Tajikistan) and US$900 (Russia) for drug-susceptible TB to ≥US$10,000 for MDR-TB (Russia). Non-TB data were scarce and inconsistent. Especially in CA, more research into the prevalence and burden of RDs is needed.

## 1. Introduction

Respiratory diseases (RDs) are common disorders in Central Asia and Russia and place a high burden on patients and health systems [1,2,3]. RDs encompass a wide range of communicable and non-communicable diseases. Tuberculosis (TB) is the most well-known communicable disease affecting the lungs, yet the majority of RDs are non-communicable. Worldwide, in 2016, an estimated 41 million deaths occurred due to non-communicable diseases (NCDs), accounting for 71% of the overall total of 57 million deaths. The deaths caused by non-communicable chronic RDs were 3.8 million, i.e., 9% of the total deaths due to NCDs [4].

The main risk factors for all types of RDs are smoking, genetics, biomass use, occupational hazards, environmental smoke exposure, lifestyle, poor nutrition, and lower socioeconomic level [5,6]. Notably, some of these risk factors are preventable traits and could be averted with early targeted preventive interventions in places where their burden is highest. These preventive measures include smoking cessation and education of patients and healthcare professionals on risk factors for RDs, such as household air pollution, and early diagnosis of RDs.

Once diagnosed, RDs such as chronic obstructive pulmonary disease (COPD), bronchiectasis, asthma, and TB require expensive and often long-term treatments, with economic consequences for both governments and patients [7,8]. To optimize governments’ budget allocation, it is of utmost importance that patients receive the most cost-effective treatments available. In most (former soviet) Central Asian countries and Russia, governments play a key role in healthcare budget allocation. In order to put RDs higher on the governments’ policy agenda and to optimally inform decision-making on healthcare spending in Central Asian countries and Russia, up-to-date and comprehensive insight into the burden of RDs in Central Asia and Russia is required.

So far, some previous individual studies and global reports have focused on RDs’ impact on mortality, yet a systematic overview of the wider clinical and economic burden of all RDs in Central Asia and Russia is lacking. Therefore, our aim was to review the prevalence and economic burden of RDs in the general population of Central Asia and Russia.

## 2. Materials and Methods

### 2.1. Study Design

We conducted a systematic review on the prevalence and economic burden of respiratory diseases in Central Asia and Russia. The study was reported according to the PRISMA checklist [9], which is provided in Supplementary Materials Table S1.

### 2.2. Protocol and Registration

The study protocol was registered at the PROSPERO website (https://www.crd.york.ac.uk/prospero/#loginpage). The registration number is CRD42018107154.

### 2.3. Eligibility Criteria

Regarding the setting and outcomes, studies should report on prevalence and/or economic burden of respiratory diseases in Central Asia and Russia. Central Asian countries include Kyrgyzstan, Uzbekistan, Tajikistan, Kazakhstan, and Turkmenistan. The patient population should comprise patients with respiratory diseases. Respiratory diseases considered were asthma, bronchiectasis, COPD, cystic fibrosis, lung cancer, pulmonary hypertension, and TB. Given the high rates of TB in the Central Asian region, we did also allow studies reporting on the prevalence of multidrug-resistant TB (MDR-TB) in TB patients. The language of the studies could be English or Russian, because global studies are mostly in English and articles in local Central Asian journals are mostly in Russian. We included full-text papers (i.e., excluding conference abstracts) of epidemiological or economic burden studies (either model-, population-, or survey-based) published in peer-reviewed journals or national reports and reviews from official governmental sources. We included all papers from the start of the databases searched until December 2018.

### 2.4. Information Sources

The databases that were searched were PUBMED and EMBASE and further snowballing on identified papers was performed through contacting authors and inspecting references. In the protocol, we also stated that, if no sufficient data would be found, we would search in the Central Scientific Medical Library in Bishkek, Kyrgyzstan. This did not turn out to be necessary given the availability of electronic records.

### 2.5. Search

In the database search, we used combinations of the following keywords (MeSH terms): “prevalence”, “burden”, “economics”, “respiratory diseases”, “COPD”, “asthma”, “tuberculosis”, “cystic fibrosis”, “interstitial lung diseases”, “lung cancer”, “bronchiectasis”, “pulmonary hypertension”, “Central Asia”, “Kazakhstan”, “Tajikistan”, “Kyrgyzstan”, “Uzbekistan”, “Turkmenistan”, and “Russia”. The full search strategy is available in Supplementary Materials Text S1.

### 2.6. Study Selection

Two reviewers (A.T. and B.E.) independently screened the two databases. A third reviewer (J.F.M.v.B.) was included in this process if any disputable issues arose. Reviewers assessed the titles and abstracts of the complete list of papers from the literature search and checked potential eligibility given the predefined inclusion and exclusion criteria. The full texts of potentially eligible papers based on title/abstract screening were read in detail and further assessed for final inclusion, using the inclusion and exclusion criteria. To aid and trace the review process, the online review program Covidence was used. Covidence enables importing citations, abstract, and full-text screening and is also a core component of Cochrane’s review production toolkit. The study selection process was reported, using the PRISMA flow diagram.

### 2.7. Data Extraction Process

A piloted and optimized data extraction form (Word document) was used to extract data from the eligible articles. The data extraction was performed by two independent reviewers (A.T. and B.E.) and checked for any discrepancies by a third reviewer (J.F.M.v.B.).

### 2.8. Data Items

The following data items were extracted from each eligible article or report: first author, study publication year, study design, sample size, country, population characteristics (age and gender distribution), disease and disease characteristics (e.g., severity), disease prevalence, and economic impact data. Prevalence and economic studies were reported separately.

### 2.9. Quality Assessment

We made a quality assessment of the articles, using the Newcastle Ottawa Scale [10] for cross-sectional studies, as reported in Supplementary Materials Table S2. This scale ranges from 0 to 10 and consists of a selection (max 5 points), comparability (max 2 points) and an outcome subsection (max 3 points).

### 2.10. Summary Measures

Principle summary measures were the prevalence of RDs and economic burden of RDs (in dollars). In this burden, we considered both direct cost (like medication and hospital admissions) and indirect cost (like work productivity losses) [11], if reported. Euros were converted to dollars at the exchange rate of the respective year when the study was conducted. If possible, subgroup data based on age, sex, and/or country were reported in a narrative manner.

### 2.11. Synthesis of Results

In the protocol, we stated that if possible, data synthesis would be performed. Given no sufficient and high-quality comparable results were available, no data synthesis was performed.

## 3. Results

### 3.1. Study Selection

**Figure 1:**
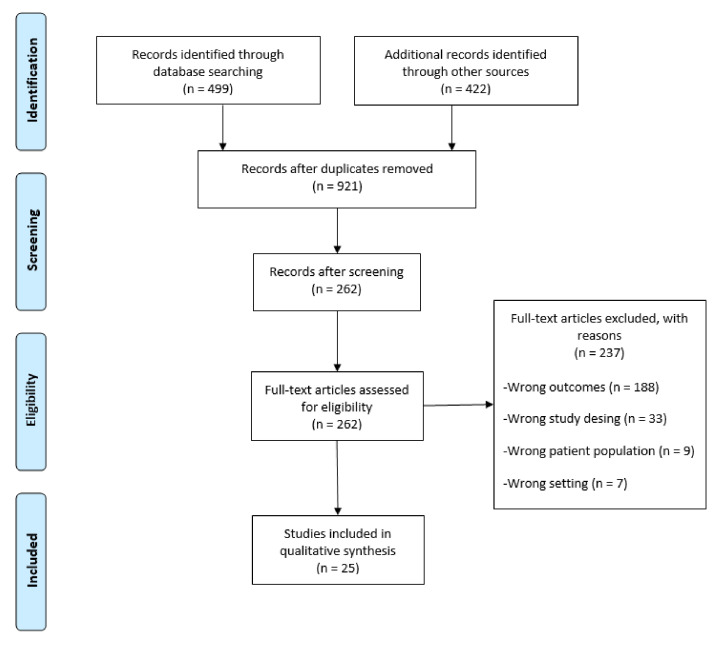
PRISMA flow diagram.

The searches in PubMed and EMBASE yielded 921 articles in total. After removing duplicates and screening of titles and abstracts, we identified 262 articles. After reviewing of the full texts, 25 articles (18 about prevalence, and seven about economic burden) meeting the inclusion criteria remained. Of these, one paper was in Russian and the remaining in English. Results of the selection process are presented in Figure 1. Details of the search results per database are provided in Supplementary Materials Text S1.

### 3.2. Prevalence of Respiratory Diseases in Central Asia and Russia

**Figure 2:**
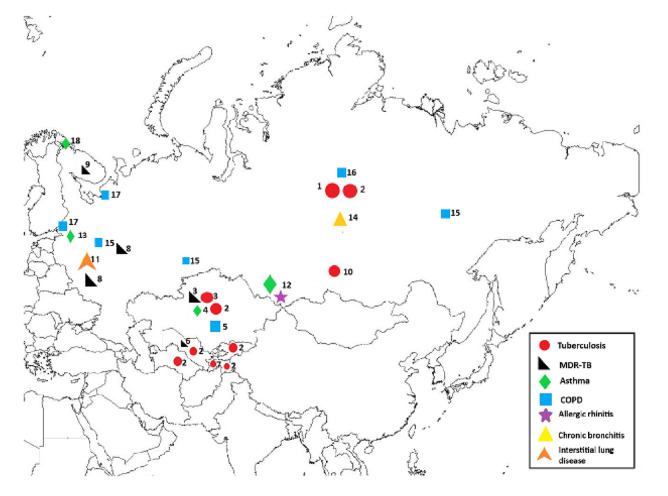
Overview of geographic locations of respiratory disease prevalence studies in Russia and Central Asia. (1) Russia (nationwide) [12]; (2) Kazakhstan, Kyrgyzstan, Tajikistan, Turkmenistan, Uzbekistan, Russia (nationwide) [13]; (3) Kazakhstan (nationwide) [14]; (4) Almaty, Kazakhstan [26]; (5) Almaty, Kazakhstan [23]; (6) Uzbekistan [16]; (7) Sughd province, Tajikistan [15]; (8) Vladimir region, Russia [17]; (9) Murmansk region, Russia [18]; (10) Krasnoyarsk region, Russia [19]; (11) Moscow, Russia [29]; (12) Altay region, Russia [28]; (13) St. Petersburg, Russia [25]; (14) Russia [20]; (15) Vologda, Chelyabinsk, Yakutsk, Russia [21]; (16) Russia [22]; (17) St. Petersburg and Arkhangelsk, Russia [24]; and (18) Nikel, Russia [27].

An overview of the geographic locations of the prevalence studies is provided in Figure 2.

The main data of all retrieved studies that reported the prevalence of respiratory diseases in Central Asia and Russia are provided in Table 1.

The table shows that, in Kyrgyzstan, Tajikistan, Turkmenistan, and Uzbekistan, there are no data on RDs, except for TB. Generally, TB prevalence declined over time, from 245 per 100,000 in 2001 in Russia to less than 100 per 100,000 across Russia and CA in 2018. Among newly treated TB patients, 19%–26% had MDR-TB, while, in previously treated TB patients, MDR-TB was present in 60%–70%. TB prevalence in a risk population of prisoners was as high as 4.5%. In the six studies on COPD, prevalence varied between 2% and 15% with the notice that different underlying populations and diagnostic methods were used. Of the four asthma (like symptoms) studies, prevalence ranged from 11% in the Russian infant population, to 5%–7% in the general Russian pediatric population, to 2% (doctor diagnosed)–25% (wheezing symptoms) in the Kazakh adult population. Moreover, here, definitions of asthma varied. Based on extrapolation of a local survey in Moscow, Russia, idiopathic pulmonary fibrosis was estimated to be present in 9–11 cases per 100,000 population (0.006%).

### 3.3. Economics of Respiratory Diseases in Central Asia and Russia

An overview of all seven studies that reported the economic consequences of RDs in Central Asia and Russia is provided in Table 2. Only two of these seven studies were not on TB. Generally, studies reported on different aspects and cost segments ranging from monthly to episodic and annual and three-year costs for TB. MDR-TB treatment was significantly higher than TB treatment. Notably, we could not find any data for cost of RDs in Turkmenistan and Uzbekistan, and no data for the cost of RDs, except for TB, for Kyrgyzstan and Kazakhstan.

### 3.4. Quality of Studies

As described earlier, we used the Newcastle Ottawa Scale for the quality assessment of the individual studies. The quality assessment is presented in Supplementary Materials Table S2. The overall quality was deemed poor. For all studies together, the total average score was 5 (SD = 1.89) out of 10. For the selection section, the mean score was 2 (SD = 1.2) out of 5, with only two studies [27,28] gaining the maximum number of points (5). In the comparability section, the mean score was 1 (SD = 0.2) out of 2. In the outcome section, the mean score was 1 (SD = 0.2) out of 3.

## 4. Discussion

### 4.1. Main Findings

We conducted a systematic literature review to identify published data on the prevalence and economics of RDs in Central Asia and Russia. Our review indicated that few studies were conducted on the prevalence (*n* = 18) and economics (*n* = 7) of RDs in this geographic area, and their quality was generally poor. The majority (74% for prevalence and 57% for economics) of the studies that were identified reported data for Russia. The rest reported data for the former Soviet states Kyrgyzstan, Tajikistan, Kazakhstan, Uzbekistan, and Turkmenistan. Regarding the type of RD, we mostly identified studies on TB, for both prevalence (*n* = 7) and economics (*n* = 5). Other studies reported on COPD, asthma, and interstitial lung diseases. No studies on cystic fibrosis, lung cancer, and pulmonary hypertension were found. TB prevalence rates in Russia and CA declined from over 200 per 100,000 20 years ago to less than 100 per 100,000 currently, but remains significantly higher in risk populations such as prisoners. MDR-TB prevalence varied from 19% to 26% in newly treated TB patients and 60% to 70% in previously treated TB patients. COPD ranged between 2% and 15%, yet different underlying populations and diagnostic methods were used. Asthma prevalence ranged between 2% and 25%, but three out of the four studies reported in pediatric populations only and definitions of asthma varied. The economic costs for TB varied between US$400 (Tajikistan) and US$900 (Russia) for drug-susceptible TB to over US$10,000 for MDR-TB in Russia. Annual COPD costs were only reported for Russia and estimated at around US$1700. For asthma, only exacerbation treatment costs in Russia were found.

### 4.2. Interpretation

In the retrieved articles, the TB prevalence varied among the different studies conducted in Russia. This can partly be explained by the different points in time in which the studies took place, but also by different geographic locations, method of data collection, underlying populations, and diagnostic methods. For instance, Shilova et al. [26] reported that 245 per 100,000 patients had active TB in Russia in 1999. Their data were collected from hospitals, where X-ray screening was done in only 57% of the total population and the quality of the bacteriology laboratory was lower compared to previous years. The importance of the underlying population was illustrated by Winetsky et.al. [14], who found that 4.5% of the prison inmates in Tajikistan had active disease, a number much higher than in the general population. In this study, all participants were male, and their living conditions involved poor heating and poor ventilation, and they shared living spaces with multiple cellmates. Terlikbaeva et al. [15] found that in 2010 in Kazakhstan, 166 per 100,000 population had TB, which is substantially higher than the 2015 figures (107 per 100,000) of Kyu et al. [13]. While there has been progress in TB treatment over the years, differences can also be attributed to study design (model versus national surveillance data). Additionally, there is both under- and over-diagnosis of TB cases in Kazakhstan because a substantial proportion of cases are identified clinically and unconfirmed by culture due to the limited access to culture and drug-susceptibility testing. Notably, despite regional differences and the use of slightly different methods, the percentage of MDR-TB in the total number of TB patients showed consistent, alarmingly high numbers with around 20–25% of MDR-TB in newly treated TB and 60%–70% in previously treated TB in both Russia and Uzbekistan.

In this review, we found that the six studies that reported on the prevalence of COPD varied widely, that is, from around 2% to 15%. We speculate that both the underlying population, region and local risk factors, the way the diagnosis was made, and underdiagnosis of COPD may play a role in explaining these differences. Artukhov et al. [19] and Chuchalin et al. [20] used stratified random cluster sampling; Gambaryan et al. [21] selected subjects from the clinics. Notably, in many Central Asian countries, due to cultural and socioeconomic circumstances, patients go to the hospital only when the condition worsens, and not when the first symptoms appear, which also affects the underdiagnosis of RDs. Differences can also occur due to different age groups of the participants included in prevalence studies, as well as the way the diagnosis was made (e.g., by self-report or by objective testing). For example, Landis et al. [22] recruited 301 participants of 40 years and older in Russia, and estimated the prevalence of COPD, based on a broad definition of self-reported physician diagnosis of COPD or chronic bronchitis or symptoms matching chronic bronchitis, at 9.2%. Artukhov et al. [19], Gambaryan et al. [21], and Chuchalin et al. [20] found that the prevalence of COPD in Russia was 2%, 15.7%, and 15.3%. Age groups were ≥18 years old, 35–64 years old, and ≥18 years old, respectively. The reason for the low rates in Artukhov et al. [19] may be that they calculated the COPD prevalence for the total population >18 years and only included patients that had an existing recorded COPD diagnosis in a database, and they did not perform their own tests, resulting in probably underdiagnosis. In contrast, participants in Chuchalin et al. [20] completed a questionnaire and then performed spirometry with bronchodilator testing (FEV_1_/FVC < 0.7) if obstruction was observed. Gambaryan et al. [21] used only a questionnaire, but this was relatively broad and only considered COPD if patients were >40 years. This makes it difficult to directly compare these data.

Four articles on asthma were found, of which three focused on pediatric asthma and one on asthma in adults. Three papers were from Russia, and one was from Kazakhstan. Nugmanova D. et al. [26] was the only study that reported asthma prevalence in Kazakhstan as doctor-diagnosed (19.5 per 1000, 2%) and as wheezing symptoms in adults (254 per 1000, 25%). Notably, this doctor-diagnosed prevalence was in between the rates in Ukraine and Azerbaijan (assessed in the same study, using the same methods), while the prevalence of wheezing symptoms was two-to-three times higher than the other two countries, suggestive of profound underdiagnosis. The three pediatric asthma studies were all performed in Russia and included different, increasing age groups. Shakhova et al. [28] included children aged three-to-six years and found 11.1% asthma-like symptoms in the Altai region, Russia. In this study they used the ISAAC questionnaire. Selnes et al. [27] found that the prevalence of pediatric asthma in children of 7–13 years was 5.1%, and they used the Bolle and Holt formulated questionnaire to assess asthma. Glushkova et al. [25] found that the prevalence of asthma in all children (0–18 years) in urban St. Petersburg, Russia, was 7.4%. For children above five years old, spirometry was used and below only survey data.

In Kyrgyzstan, Uzbekistan, Tajikistan, and Turkmenistan, there were no data on the prevalence of asthma, COPD, interstitial lung diseases, lung cancer, and cystic fibrosis. We speculate that this is partly due to the low level of equipment and diagnostic methods, lower levels of research resource capacity and funding, and the suboptimal level of knowledge of doctors about these diseases. Indeed, TB remains the main priority RD of attention. This was also the only disease for which economic data were available, yet data were not consistent and require more robust methods of evaluation. Of note, we identified relatively many papers on TB; however, they did not clearly differentiate between acute TB and post-TB lung disease, they mainly included acute TB. We should however acknowledge that, due to the long-term nature of post-TB lung disease, the total clinical and economic burden could be even higher.

### 4.3. Strengths and Limitations

A strength of this study is that it is the first systematic review on the prevalence and economic burden focusing specifically on RDs in Central Asia and Russia. Given many of these diseases share common risk factors and have overlapping diagnostic procedures, we chose a broad range of different RDs. Notably, the comprehensive literature review was performed by two independent reviewers and reported according to PRISMA guidance. Moreover, the protocol for this study was preregistered in PROSPERO, and we used papers from peer-reviewed journals in English and Russian languages. We used for quality assessment the Newcastle Ottawa Scale. A limitation of this review was that we did not include papers from local Republic Scientific Medical Libraries, Government reports, and Universities, yet given their widespread geographic locations and paper-based files, these were difficult to retrieve and assess. It may, however, have caused a more selective inclusion of data that were published in the international literature.

### 4.4. Recommendations for Future Research and Policy

Given only few studies on RDs in Central Asia were found, more high-quality prevalence and economic cost studies on RDs in this area are clearly required. While some studies reported on TB, especially for COPD, asthma, interstitial lung diseases, cystic fibrosis, lung cancer, bronchiectasis, and pulmonary hypertension more data are urgently needed. In particular, data from these studies should help policymakers to promote and create programs to improve early diagnosis and prevention of RDs in order to reduce their clinical patient burden, as well as costs for governments and patients. Moreover, local clinical guidelines on these diseases should better reflect local resources, epidemiology, risk factors, burden, and implementation capacity [36].

It is important to note that the situation on TB in CA remains a significant problem. Although the absolute number of patients has decreased, still, compared to Europe and America, the prevalence of TB is extremely high, placing enormous costs on the government and patients. This is partly due to the socioeconomic level of the population, but also the general understaffing of healthcare facilities. For example, in Kyrgyzstan, there is one doctor and one nurse per village. As there is no equipment in the villages, severe and unclear patients are referred to central district hospitals, where one doctor serves up to 20–30 patients per day, which can also affect the quality of medical care. Again, this calls for smart priority setting and cost-effective resource allocation, informed by proper epidemiologic studies, to eventually reduce the burden of RDs.

## 5. Conclusions

This review showed that most studies on RDs prevalence and economics in Russia and Central Asia were performed on TB. Generally, besides TB data, data on other RDs in Central Asia are scarce, low-quality, and inconsistent. To further guide health policy, more research, especially in Central Asia, is needed to clarify the prevalence and economic costs of RDs in this area.

## Figures and Tables

**Table 1:** Studies providing prevalence data of respiratory disease in Central Asia and Russia (*n* = 18).

Study (1st Author, Year)	Study Design	Sample Size	Country/Region	Population Characteristics (Age, Gender)	Disease and Disease Characteristics (e.g., Severity)	Disease Prevalence
Shilova et al. [12], 2001	Retrospective study	2,512,300	Russia	Not specified	TB	245 per 100,000
Kyu et al. [13], 2018	Model based study	Total national populations	Kazakhstan, Kyrgyzstan, Tajikistan, Turkmenistan, Uzbekistan, Russia	Not specified	TB	* Kazakhstan: 107 per 100,000; Kyrgyzstan: 66 per 100,000; Tajikistan: 54 per 100,000; Turkmenistan: 84 per 100,000; Uzbekistan: 66 per 100,000; Russia: 81 per 100,000
Terlikbaeva et al. [14], 2012	Epidemiological study	Total national population	Kazakhstan	Not reported	TB and MDR-TB	TB: 166.3 per 100,000 (0.16%) MDR-TB: 61.6 per 100,000 (0.06%)
Winetsky et al. [15], 2014	Cross-sectional study	1317	Sughd province, Tajikistan	Prison population Mean age: 36 Male: 100%	Pulmonary TB	4.5%
Ulmasova et al. [16], 2013	Country wide survey	1037	Uzbekistan	Mean age: 60% under 45 years Male: 56%	MDR-TB in TB patients	MDR-TB (new cases): 23.2% MDR-TB (previously treated): 62%
Punga et al. [17], 2009	Cross-sectional retrospective survey	1882	Vladimir and Orel regions, Russia	Mean age: 44 Male: 21%	MDR-TB in TB patients	MDR-TB Vladimir: 19% Orel: 20%
Mäkinen et al. [18], 2011	Population-based survey	1226	Murmansk region, Russia	Not reported	MDR-TB in TB	MDR-TB (new cases): 26%, MDR-TB (previously treated): 72.9%
Artyukhov et al. [19], 2015	Population-based epidemiological study	15,000	Krasnoyarsk region, Russia	Age group: 18 and older	COPD	COPD: 21.2 per 1000 (2%) inhabitants
Chuchalin et al. [20], 2014	Cross-sectional population-based epidemiological study	7164	Russia	Mean age: 43.4 Female: 57.2%	Asthma related symptoms, chronic bronchitis, COPD	Asthma related symptoms: 25.7% chronic bronchitis: −8.6%; COPD (extrapolated): 15.3%
Gambaryan et al. [21], 2017	Cross-sectional epidemiological study	3771	Vologda, Chelyabinsk, Yakutsk, Russia	Mean age: 48.8 Male: 36.9% Female: 63.1%	COPD	Males: 14.7–12.9%, Females: 15.7%
Landis et al. [22], 2014	Population-based study	4343	Russia	Age group: 40–70+ Male: 39%	COPD	Russia, Overall: 9.2%, Males: 11.4%, Females: 8.3%
Nugmanova et al. [23], 2018	Population-based cross-sectional study	945	Almaty, Kazakhstan	Mean age: 42.5 (SD: 15.3) Male: 36.8% Female: 63.2%	COPD	66.7 per 1000 (6.7%)
Andreeva et al. [24], 2016	Population-based cross-sectional study	3133	St. Petersburg and Arkhangelsk, Russia	Mean age: 54 (SD: 9.25) Male: 31.8%	COPD	6.8%
Glushkova et al. [25], 2008	Cross-sectional study	1464	St. Petersburg, Russia	Age group: 0–18 Boys: 8.5% Girls: 6.2%	Asthma	7.4%
Nugmanova et al. [26], 2018	Cross-sectional population-based epidemiological study	945	Almaty, Kazakhstan	Mean age: 42.5 (SD 15.3) Male: 36.8% Female: 63.2%	Bronchial asthma	Doctor diagnosed: 19.5 per 1000 (2%); wheezing symptoms: 254.8 per 1000 (25%)
Selnes et al. [27], 2001	Cross-sectional study	1143	Nikel, Russia	Age group: 7–13; Boys: 50%	Asthma	Pediatric asthma: 5.1%
Shakhova et al. [28], 2017	Cross-sectional study	3205	Altay region, Russia	Age group: 3–6; Boys: 48.9%	Asthma-like symptoms and allergic rhinitis	Asthma-like symptoms: 11.1%, allergic rhinitis: 18%
Richeldi et al. [29], 2015	Narrative review (extrapolation of survey)	Not reported	Moscow, Russia	Mean age: 60 Male: 66%	Idiopathic pulmonary fibrosis	9–11 cases per 100,000 population (0.006%)

* Absolute numbers were converted to cases per 100,000 population (based on World Bank, 2015). COPD = chronic obstructive pulmonary disease; MDR-TB = multidrug-resistant tuberculosis.

**Table 2:** Studies providing cost data of respiratory disease in Central Asia and Russia (*n* = 7).

Study (1st Author, Year)	Study Design	Sample Size	Country	Population Characteristics (Age, Gender)	Disease and Disease Characteristics (e.g., Severity)	Economic Impact Data
Atun et al. [30], 2006	Retrospective cohort study	2682	Russia	Mean age females: 38.2 and males: 40.7 Female: 20% Male: 80%	TB	The mean cumulative cost of treating a TB case over 3 years was estimated at US$886.
Ayé et al. [31], 2011	Epidemiological study	282	Tajikistan	Age group: 15–45+	TB	Mean costs for an episode of TB was US$396.
Skordis-Worrall et al. [32], 2017	Cross-sectional study	309	Kyrgyzstan	Mean age: 30.28 (12.79) Female: 58.3%	TB	Monthly costs for TB were US$6.
van den Hof et al. [33], 2016	Cross-sectional study	54 TB and 94 MDR-TB	Kazakhstan	Age group: 21–50+	TB and MDR-TB	Median costs of DS-TB treatment: US$ 929; MDR-TB treatment: US$ 3125.
Floyd et al. [34], 2012	Retrospective cohort study	124	Tomsk oblast, Russia	Mean age: 38 Male: 70% Female: 30%	MDR-TB	Annual per patient cost: US$10,088.
Foo et al. [6], 2016	Cross-sectional, population-based survey	4343	Russia	Mean age: 57	COPD stage I: 26%, stage II: 67%, stage III-IV: 7%	Annual societal cost: US$1721 (direct cost: US$742; indirect cost: US$979).
Lane et al. [35], 2006	Observational study	100	Russia	Mean age: 51.1 (SD 14.1) Male: 34%	Asthma (exacerbation)	Direct costs in primary care: €219 (US$^288.9), Secondary care: €693 (US$^914.3).

COPD—chronic obstructive pulmonary disease; TB—tuberculosis; MDR-TB—multidrug-resistant tuberculosis; ^ Euro to dollar conversion for 2006.

## Supplementary Materials

The following are available online at https://www.mdpi.com/1660-4601/17/20/7483/s1. Table S1: PRISMA-checklist. Text S1: Full search strategy. Table S2: Quality assessment.
