# Supplementary material for: Prevalence and Economic Burden of Respiratory Diseases in Central Asia and Russia: A Systematic Review
Source: Int J Environ Res Public Health. 2020 Oct 14;17(20):7483. doi: 10.3390/ijerph17207483 (PMC7602368; doi:10.3390/ijerph17207483)
Supplement: Supplementary file 1 [file ijerph-17-07483-s001.pdf]

Prevalence and Economic Burden of Respiratory Diseases in Central Asia and Russia: A Systematic Review

Supplementary Material

Table S1. PRISMA checklist. [1]

Section/topic	#	Checklist item	Reported on page #
		**TITLE**	
Title	1	Identify the report as a systematic review, meta-analysis, or both.	1
		**ABSTRACT**	
Structured summary	2	Provide a structured summary including, as applicable: background; objectives; data sources; study eligibility criteria, participants, and interventions; study appraisal and synthesis methods; results; limitations; conclusions and implications of key findings; systematic review registration number.	2
		**INTRODUCTION**	
Rationale	3	Describe the rationale for the review in the context of what is already known.	3
Objectives	4	Provide an explicit statement of questions being addressed with reference to participants, interventions, comparisons, outcomes, and study design (PICOS).	3
		**METHODS**	
Protocol and registration	5	Indicate if a review protocol exists, if and where it can be accessed (e.g., Web address), and, if available, provide registration information including registration number.	4
Eligibility criteria	6	Specify study characteristics (e.g., PICOS, length of follow-up) and report characteristics (e.g., years considered, language, publication status) used as criteria for eligibility, giving rationale.	4
Information sources	7	Describe all information sources (e.g., databases with dates of coverage, contact with study authors to identify additional studies) in the search and date last searched.	4
Search	8	Present full electronic search strategy for at least one database, including any limits used, such that it could be repeated.	4
Study selection	9	State the process for selecting studies (i.e., screening, eligibility, included in systematic review, and, if applicable, included in the meta-analysis).	4
Data collection process	10	Describe method of data extraction from reports (e.g., piloted forms, independently, in duplicate) and any processes for obtaining and confirming data from investigators.	5
Data items	11	List and define all variables for which data were sought (e.g., PICOS, funding sources) and any assumptions and simplifications made.	5
Risk of bias in individual studies	12	Describe methods used for assessing risk of bias of individual studies (including specification of whether this was done at the study or outcome level), and how this information is to be used in any data synthesis.	5
Summary measures	13	State the principal summary measures (e.g., risk ratio, difference in means).	5
Synthesis of results	14	Describe the methods of handling data and combining results of studies, if done, including measures of consistency (e.g., I^2) for each meta-analysis.	5
Section/topic	#	Checklist item	Reported on page #
Risk of bias across studies	15	Specify any assessment of risk of bias that may affect the cumulative evidence (e.g., publication bias, selective reporting within studies).	5
Additional analyses	16	Describe methods of additional analyses (e.g., sensitivity or subgroup analyses, meta-regression), if done, indicating which were pre-specified.	

**RESULTS**			
Study selection	17	Give numbers of studies screened, assessed for eligibility, and included in the review, with reasons for exclusions at each stage, ideally with a flow diagram.	6
Study characteristics	18	For each study, present characteristics for which data were extracted (e.g., study size, PICOS, follow-up period) and provide the citations.	6
Risk of bias within studies	19	Present data on risk of bias of each study and, if available, any outcome level assessment (see item 12).	10
Results of individual studies	20	For all outcomes considered (benefits or harms), present, for each study: (a) simple summary data for each intervention group (b) effect estimates and confidence intervals, ideally with a forest plot.	
Synthesis of results	21	Present results of each meta-analysis done, including confidence intervals and measures of consistency.	
Risk of bias across studies	22	Present results of any assessment of risk of bias across studies (see Item 15).	9,10
Additional analysis	23	Give results of additional analyses, if done (e.g., sensitivity or subgroup analyses, meta-regression [see Item 16]).	
**DISCUSSION**			
Summary of evidence	24	Summarize the main findings including the strength of evidence for each main outcome; consider their relevance to key groups (e.g., healthcare providers, users, and policy makers).	10
Limitations	25	Discuss limitations at study and outcome level (e.g., risk of bias), and at review-level (e.g., incomplete retrieval of identified research, reporting bias).	13
Conclusions	26	Provide a general interpretation of the results in the context of other evidence, and implications for future research.	13
**FUNDING**			
Funding	27	Describe sources of funding for the systematic review and other support (e.g., supply of data); role of funders for the systematic review.	13

Text S1 Full search strategy

Period of performing searches: September 2018

Search limits

Studies in humans

English and Russian language

Published in peer reviewed journals

Full text available

Keywords

Disease terms

“COPD” OR “chronic obstructive pulmonary disease” OR “respiratory diseases” OR “chronic bronchitis” OR “asthma” OR “tuberculosis” OR “TB” OR “cystic fibrosis” OR “interstitial lung diseases” OR “lung cancer” OR “bronchiectasis” OR “pulmonary hypertension” OR “emphysema”

Country terms

“Kyrgyzstan” OR “Kazakhstan” OR “Turkmenistan” OR “Tajikistan” OR “Uzbekistan” OR “Russia” OR “Russian Federation”

Outcome terms

“Prevalence” OR “burden” OR “economic” OR “healthcare costs” OR “costs” OR “expenditures” OR “spending”

Combined

(“COPD” OR “Chronic obstructive pulmonary disease” OR “Respiratory diseases” OR “Chronic bronchitis” OR “Asthma” OR “Tuberculosis” OR “TB” OR “Cystic fibrosis” OR “Interstitial lung diseases” OR “Lung cancer” OR “Bronchiectasis” OR “Pulmonary hypertension” OR “Emphysema”) AND (“Kyrgyzstan” OR “Kazakhstan” OR “Turkmenistan” OR “Tajikistan” OR “Uzbekistan” OR “Russia” OR “Russian Federation”) AND (“Prevalence” OR “Burden” OR “Economic” OR “Healthcare costs” OR “Costs” OR “Expenditures” OR “Spending”)

Search strategy

1. Search disease terms
2. Search country terms
3. Search outcomes terms
4. Combined disease, country and outcome terms
5. Exclude reviews, editorials and other non-research articles
6. Screen titles and abstract for studies on the prevalence and economic burden of respiratory diseases in Central Asia and Russia

PubMed search results

1. Disease terms: 752918
2. Country terms: 135858
3. Outcomes terms: 1170304
4. Combined terms: 667
5. Combined terms and humans and published from 2000: 499
6. Screening results: 323 agreed, (Yes: 163), 176 resolve conflicts, 191 full articles we tried to find full text for 109 we could not find in PubMed /

Google / researchgate and for those the authors were contacted, from 109 we did not received the full text. All full text articles the total was 82.

7. Combined terms and full text available: 283
8. Articles excluded: 179 *Reasons:* wrong outcomes = 139, wrong study design =26, wrong setting = 6, wrong patient population = 9.
9. Final selection: 12
10. Contact authors: 83

EMBASE search results

1. Disease terms: 1304605
2. Country terms: 730492
3. Outcomes terms: 1632867
4. Combined terms: 1580
5. Combined terms and full text available: 416 (We tick the filters: *Date:* from 2000 till 2018. *Sources:* EMBASE. *Publication type:* article. *Quick limits:* Human. *Languages:* English and Russian. *Publication types:* article.)
6. Screening results: 422 agreed, (Yes: 129), 106 resolve conflicts, 91 full articles we tried to find full text for 12 we could not find in PubMed / Google / researchgate and for those the authors were contacted, from 10 we did not receive the full text. All full text articles the total was 79.
7. Articles excluded: 56 *Reasons:* wrong outcomes = 49, wrong study design = 7, wrong setting = 1.

Final selection: 23

Table S2. Quality assessment.

Study (1st author, year)	Selection (max. 5*)	Comparability (max: 2*)	Outcome (max. 3*)	Total (max: 10*)
Artyukhov I.P. et al.[2], 2015	***	**	*	6
Chuchalin A.G. et al.[3], 2014	****	*	**	7
Gambaryan M.H. et al.[4], 2017	**	*		3
Glushkova A.V. et al.[5], 2008	***	*	**	6
Kyu H.H. et al.[6], 2018	**	*	*	4
Landis S.H. et al.[7], 2014	***	*	**	6
Mäkinen J. et. al.[8], 2011	****	*	***	8
Nugmanova D. et al,[9] 2018	****	*	**	7
Nugmanova D. et al.[10], 2018	****	*	**	7
Punga V.V. et. al.[11], 2009	*	*	**	4
Richeldi L. et. al.[12], 2015			*	1 (#)
Selnes A. et al.[13], 2001	*****	*	*	7
Shakhova N. et al.[14], 2017	*****	*	**	8
Ulmasova D.J. et. al.[15], 2013	***	*	***	7
Winetsky D.E. et. al.[16], 2014	***	*	*	5
Andreeva E. et al.[17], 2016	***	*	**	6
Shilova M. et al.[18], 2001	**	*	**	5
Terlikbaeva et al.[19], 2012	**	*	**	5
Atun R.A. et. al.[20], 2006	**	*	**	5
van den Hof S. et al.[21],	*		**	3

2016				
Ayé R. et al.[22], 2011	*	*	**	4
Foo J. et. al.[23], 2016	****	*	***	8
Floyd K. et al.[24], 2012	**	*	***	6
Lane S. et al.[25], 2006	*	*		2
Skordis-Worrall J. et al.[26], 2017	**	*	**	5
Mean (SD)	5 (1.87)	2 (1.2)	1 (0.2)	2 (0.6)

* Star, where more stars mean higher quality; # note that this was a narrative review, so this score cannot be interpreted as a measure of quality.

Reference list Supplementary materials

1. PRISMA. Preferred Reporting Items of Systematic Reviews and Meta-Analyses. Available online: http://prisma-statement.org (accessed on 5 March 2020).

2. Artyukhov, I.; Arshukova, I.; Dobretsova, E.; Dugina, T.; Shulmin, A.; Demko, I. Epidemiology of chronic obstructive pulmonary disease: A population-based study in Krasnoyarsk region, Russia. *Int. J. Chronic Obstr. Pulm. Dis*. **2015**, 1781–1786.

3. Chuchalin, A.G.; Khaltaev, N.; Antonov, N.S.; Galkin, D.V.; Manakov, L.G.; Antonini, P.; Demko, I.V. Chronic respiratory diseases and risk factors in 12 regions of the Russian Federation. *Int. J. Chronic Obstr. Pulm. Dis*. **2014**, 9, 963–974.

4. Gambaryan, M.H.; Shalnova, S.A.; Deev, A.D.; Drapkina, O.M. Chronic Respiratory Diseases in the Regions of Northern Russia: Epidemiological Distinctions in the Results of a National Population Study. *Int. J. Environ. Res. Public Health* **2017**, 14, 841.

5. Glushkova, A.V.; Grjibovski, A.M. Prevalence and correlates of asthma among children in central St. Petersburg, Russia: Cross-sectional study. *Croat. Med. J.* **2008**, 49, 741–750.

6. Kyu, H.H.; Maddison, E.R.; Henry, N.J. The global burden of tuberculosis: Results from the Global Burden of Disease Study 2015. *Lancet. Infect. Dis.* **2018**, *18*, 261–284.

7. Landis, S.H.; Muellerova, H.; Mannino, D.M.; Menezes, A.M.; Han, M.K.; van der Molen, T.; Davis, K.J. Continuing to Confront COPD International Patient Survey: Methods, COPD prevalence, and disease burden in 2012–2013. *Int. J. Chronic Obstr. Pulm. Dis.***2014**, *9*, 597–611.

8. Mäkinen, J.; Marjamäki, M.; Haanperä-Heikkinen, M.; Marttila, H.; Endourova, L.B.; Presnova, S.E.; Mathys, V.; Bifani, P.; Ruohonen, R.; Viljanen, M.K.; et al. Extremely high prevalence of multidrug resistant tuberculosis in Murmansk, Russia: A population-based study. *Eur. J. Clin. Microbiol. Infect. Dis.* **2011**, 30, 1119–1126.

9. Nugmanova, D.; Sokolova, L.; Feshchenko, Y.; Iashyna, L.; Gyrina, O.; Malynovska, K.; Tariq, L. The prevalence, burden and risk factors associated with bronchial asthma in commonwealth of independent states countries (Ukraine, Kazakhstan and Azerbaijan): Results of the CORE study. *BMC Pulm. Med.* **2018**, 18, 110.

10. Nugmanova, D.; Feshchenko, Y.; Iashyna, L.; Gyrina, O.; Malynovska, K.; Mammadbayov, E.; Vasylyev, A. The prevalence, burden and risk factors associated with chronic obstructive pulmonary disease in Commonwealth of Independent States (Ukraine, Kazakhstan and Azerbaijan): Results of the CORE study. *BMC Pulm. Med.* **2018**, 18, 26.

11. Punga, V.V.; Jakubowiak, W.M.; Danilova, I.D.; Somova, T.R.; Volchenkov, G.V.; Kazionnyy, B.Y.; Nemtsova, E.S.; Kiryanova, E.V.; Kourbatova, E.V. Prevalence of extensively drug-resistant tuberculosis in Vladimir and Orel regions, Russia. *Int. J. Tuberc. Lung Dis.* **2009**, 3, 1309–1312.

12. Richeldi, L.; Rubin, A.S.; Avdeev, S.; Udwadia, Z.F.; Xu, Z.J. Idiopathic pulmonary fibrosis in BRIC countries: The cases of Brazil, Russia, India, and China. *BMC Med*. **2015**, 13, 237.

13. Selnes, A.; Odland, J.Ø.; Bolle, R.; Holt, J.; Dotterud, L.K.; Lund, E. Asthma and allergy in Russian and Norwegian schoolchildren: Results from two questionnaire-based studies in the Kola Peninsula, Russia, and northern Norway. *Allergy* **2001**, 56, 344–348.

14. Shakhova, N.V.; Kamaltynova, E.M.; Lobanov, Y.F.; Ardatova, T.S.; Nikolaeva, K.S. The prevalence and risk factors of allergic rhinitis among the children of the preschool age. *Vestn. Otorinolaryngol*. **2017**, 82, 47–51.

15. Ulmasova, D.J.; Uzakova, G.; Tillyashayhov, M.N.; Turaev, L.; van Gemert, W.; Dara, M.; Kremer, K.; Gombogaram, T.; Dara, M.; du Cros, P.; et al. Multidrug-resistant tuberculosis in Uzbekistan: Results of a nationwide survey, 2010 to 2011. *Eurosurveillance* **2013**, 18, 42.

16. Winetsky, D.E.; Almukhamedov, O.; Pulatov, D.; Vezhnina, N.; Dooronbekova, A.; Zhussupov, B. Prevalence, risk factors and social context of active pulmonary tuberculosis among prison inmates in Tajikistan. *PLoS ONE* **2014**, 9, e86046.

17. Andreeva, E.; Pokhaznikova, M.; Lebedev, A.; Moiseeva, I.; Kutznetsova, O.; Degryse, J.M. The Prevalence of Chronic Obstructive Pulmonary Disease by the Global Lung Initiative Equations in North-Western Russia. *Respiration* **2016**, 91, 43–55.

18. Shilova, M.V.; Dye, C. The resurgence of tuberculosis in Russia. *Philos. Trans. R. Soc. Lond. Ser. B Biol. Sci.* **2001**, 356, 1069–1075.

19. Terlikbayeva, A.; Hermosilla, S.; Galea, S.; Schluger, N.; Yegeubayeva, S.; Abildayev, T.; El-Bassel, N. Tuberculosis in Kazakhstan: Analysis of risk determinants in national surveillance data. *BMC Infect. Dis.* **2012**, 12, 262.

20. Atun, R.A.; Samyshkin, Y.; Drobniewski, F.; Balabanova, Y.; Fedorin, I.M.; Lord, J.; Coker, R.J. Costs and outcomes of tuberculosis control in the Russian Federation: Retrospective cohort analysis. *Health Policy Plan*. **2006**, 21, 353–365.

21. van den Hof, S.; Collins, D.; Hafidz, F.; Beyene, D.; Tursynbayeva, A.; Tiemersma, E. The socioeconomic impact of multidrug resistant tuberculosis on patients: Results from Ethiopia, Indonesia and Kazakhstan. *BMC Infect. Dis*. **2016**, 16, 470.

22. Ayé, R.; Wyss, K.; Abdualimova, H.; Saidaliev, S. Factors determining household expenditure for tuberculosis and coping strategies in Tajikistan. *Trop. Med. Int. Health* **2011**, 16, 307–313.

23. Foo, J.; Landis, S.H.; Maskell, J.; Oh, Y.M.; van der Molen, T.; Han, M.K.; Mannino, D.M.; Ichinose, M.; Punekar, Y. Continuing to Confront COPD International COPD Survey: Economic impact of COPD in 12 counties. *PLoS ONE* **2016**, 11, e0152618.

24. Floyd, K.; Hutubessy, R.; Kliiman, K.; Centis, R.; Khurieva, N.; Jakobowiak, W.; Danilovits, M.; Peremitin, G.; Keshavjee, S.; Migliori, G.B. Cost and cost-effectiveness of multidrug-resistant tuberculosis treatment in Estonia and Russia. *Eur. Respir. J*. **2012**, 40, 133–142.

25. Lane, S.; Molina, J.; Plusa, T. An international observational prospective study to determine the Cost Of Asthma eXacerbations (COAX). *Respir. Med*. **2006**, 100, 434–450.

26. Skordis-Worrall, J.; Round, J.; Arnold, M.; Abdraimova, A.; Akkazieva, B.; Beran, D. Addressing the double-burden of diabetes and tuberculosis: Lessons from Kyrgyzstan. *Glob. Health* **2017**, 13, 16.
